# Integrating Baseline Nutritional and Inflammatory Parameters with Post-Treatment EBV DNA Level to Predict Outcomes of Patients with De Novo Metastatic Nasopharyngeal Carcinoma Receiving Chemotherapy Combination PD-1 Inhibitor

**DOI:** 10.3390/nu15194262

**Published:** 2023-10-05

**Authors:** Jia Guo, Qi Yang, Qi Jiang, Li-Wen Gu, Huan-Xin Lin, Ling Guo

**Affiliations:** 1 State Key Laboratory of Oncology in South China, Guangdong Key Laboratory of Nasopharyngeal Carcinoma Diagnosis and Therapy, Guangdong Provincial Clinical Research Center for Cancer, Sun Yat-sen University Cancer Center, Guangzhou 510060, China; guojia@sysucc.org.cn (J.G.); yangqi@sysucc.org.cn (Q.Y.); jiangqi@sysucc.org.cn (Q.J.); gulw@sysucc.org.cn (L.-W.G.); linhx@sysucc.org.cn (H.-X.L.); 2 Department of Nasopharyngeal Carcinoma, Sun Yat-sen University Cancer Center, Guangzhou 510060, China; 3 Department of Radiation Oncology, Sun Yat-sen University Cancer Center, Guangzhou 510060, China

**Keywords:** nasopharyngeal carcinoma, nutritional status, inflammation, PD-1 inhibitor, prognostic model

## Abstract

Objectives: To develop and validate a prognostic nomogram based on baseline nutritional and inflammatory parameters for risk stratification in patients with de novo metastatic nasopharyngeal carcinoma (dmNPC) receiving chemotherapy combination programmed death-1 (PD-1) inhibitor. Methods: This retrospective study analyzed 131 patients with dmNPC (88 and 43 in the training and validation cohorts, respectively) between March 2017 and November 2020. All these patients received chemotherapy combined with PD-1 inhibitor treatment. We identified independent risk factors using univariate and multivariate Cox regression analyses and established a nomogram to predict the progression-free survival (PFS). The predictive accuracy of the nomogram was evaluated and independently validated. Results: Baseline nutritional risk index (NRI), prognostic nutritional index (PNI), systemic immune-inflammation index (SII), uric acid (UA), and post-treatment Epstein–Barr virus (EBV) DNA were used to develop a nomogram that could divide patients into favorable- and unfavorable-prognosis groups. The median PFS (mPFS) was significantly longer in the favorable-prognosis group compared to the unfavorable-prognosis group (35.10 months [95% CI: 27.36–42.84] vs. 7.23 months [95% CI: 6.50–7.97]; *p* = 0.001). All results were confirmed in the validation cohort. Conclusions: The proposed model improved the prognostic risk stratification for patients with dmNPC undergoing chemotherapy combined with PD-1 inhibitor treatment.

## 1. Introduction

Nasopharyngeal carcinoma (NPC) is one of the most common head and neck cancers and is prevalent in Southern China, North Africa, and Southeast Asia [1]. At the time of initial diagnosis, approximately 4–10% of patients exhibit distant metastasis, resulting in a diagnosis of de novo metastatic nasopharyngeal carcinoma (dmNPC). Patients who are diagnosed with dmNPC typically have a median overall survival (OS) of approximately 10–36 months [2]. In recent years, the combination of platinum-based systemic chemotherapy and programmed death-1 (PD-1) inhibitors has emerged as the preferred first-line treatment for dmNPC [3]. However, the efficacy of this treatment varies among patients with dmNPC. Considering such heterogeneity in prognosis, individualized treatment and surveillance strategies are of utmost importance to maximize the survival benefit. Patients with an unfavorable prognosis should receive more intense therapy and be closely monitored. However, no easy-to-use biomarkers are available in prognosis stratification for patients with dmNPC after chemotherapy combination PD-1 inhibitor treatment.

Numerous studies have been conducted to investigate the impact of nutritional status on the prognosis of cancer patients. Patients who are malnourished exhibit reduced tolerance to the adverse reactions of antitumor drugs, thus leading to inadequate responses to chemotherapy and unfavorable outcomes [4]. Various nutritional indicators, such as the nutritional risk index (NRI), prognostic nutritional index (PNI), and controlling nutritional status (CONUT) scores, have been found to have strong associations with therapeutic responses, survival outcomes, and treatment-related complications in patients with NPC [5,6,7]. However, data on the prognostic value of the nutritional indicators for patients with dmNPC who underwent combined chemoimmuocherapy are still unavailable. Moreover, which nutritional index has superior predictive power remains unclear.

In addition to nutritional status, emerging evidence suggests a close relationship between the initiation, progression, invasion, and metastasis of NPC and the inflammatory microenvironment within the tumor [8]. Several inflammatory biomarkers, including the systemic immune-inflammation index (SII), systemic inflammatory response index (SIRI), platelet-to-lymphocyte ratio (PLR), and neutrophil-to-lymphocyte ratio (NLR), have been demonstrated as effective prognostic factors in various types of cancer, representing diverse inflammatory and immune pathways in vivo [9,10,11]. Nevertheless, no reliable inflammatory biomarker is currently available to predict the efficacy of chemoimmunotherapy in patients with dmNPC.

However, the absence of tumor-related factors makes it unreliable to rely solely on nutritional or inflammatory parameters for predicting the outcomes of dmNPC. Plasma Epstein–Barr virus (EBV) deoxyribonucleic acid (DNA) level, one of the most dependable prognostic indicators for NPC, have been widely used for disease screening, prognosis evaluation, and monitoring of disease progression [12,13,14]. EBV-DNA titer has proven to be a valuable biomarker for predicting prognosis and monitoring disease progression in patients with RM-NPC undergoing immunotherapy [15]. Unfortunately, the time point at which the EBV DNA level measured could better reflect the tumor burden remains uncertain. Furthermore, an optimal cut-off value of plasma EBV DNA levels is still being determined.

To the best of our knowledge, no validated nutritional or inflammatory parameters exist that are capable of predicting the outcomes of patients with dmNPC who have been treated with chemotherapy combined with PD-1 inhibitor. Thus, this study is aimed at clarifying the prognostic and predictive values of the nutritional indicators, inflammatory biomarkers, and plasma EBV DNA level to predict survival among patients with dmNPC. Additionally, we established and validated a novel predictive model based on a combination of baseline nutritional, inflammatory, and other clinical indicators to optimize risk stratification of patients with dmNPC and to appropriately determine treatment and surveillance strategies following chemotherapy in combination with a PD-1 inhibitor.

## 2. Materials and Methods

### 2.1. Data Extraction and Study Population

Figure S1 illustrates a flowchart depicting the patient enrollment strategy and inclusion criteria. A total of 131 patients who were diagnosed with de novo metastatic NPC and received a first-line or subsequent-line chemotherapy combination PD-1 inhibitor in our institution between March 2017 and November 2020 were included in this study. Patients were excluded from the study if they had (i) a history of previous or synchronous malignant tumors (n = 19), (ii) asynchronous metastasis after curative treatment (n = 52), (iii) received less than two cycles of chemotherapy in combination with a PD-1 inhibitor (n = 30), or (iv) incomplete clinical data (n = 87). Patients were randomly assigned to either the training cohort (n = 88) or the validation cohort (n = 43) in a 2:1 ratio for model development and verification. The staging of all patients was determined using the 8th edition of the AJCC staging system. The study was approved by the Ethics Committee of our institution (approved number: B2023-492-01). Informed consent was waived due to the retrospective nature of the study and the anonymization of the patients’ data.

### 2.2. Data Collection and Classification

Candidate nutritional and inflammatory parameters were selected to develop a nomogram after reviewing the literature. This study focused on 18 indexes, including NRI, PNI, SII, SIRI, LMR, PLR, NLR, COUNT score, Glasgow prognostic score (GPS), lactate dehydrogenase-to-albumin ratio (LAR), lactate dehydrogenase (LDH) levels, gamma-glutamyl transferase (GGT) levels, C-reactive protein (CRP) levels, alkaline phosphatase (ALP) levels, uric acid (UA) levels, glucose (GLU) levels, creatine kinase (CK) levels, and serum amyloid A (SAA) levels. The levels of LDH, GGT, CRP, ALP, UA, GLU, CK, and SAA were obtained from routine laboratory examination results. EBV DNA concentrations were measured before and after treatment at diagnosis and within one week after completing chemotherapy, following the methods described in previous studies [16]. Other indicators were calculated according to the following formulas: NRI = 1.487 × albumin (g/L) + 41.7 × weight/ideal body weight (kg), where ideal body weight was defined as 22 × height (m)^2^ [17]; PNI = albumin (g/L) + 5 × lymphocyte counts (10^9^/L) [18]; NLR, LAR, LMR, and PLR as the ratios of the absolute neutrophil count (10^9^/L) to the absolute lymphocyte count (10^9^/L), lactate dehydrogenase (U/L) to albumin (g/L), the absolute lymphocyte count (10^9^/L) to the absolute monocyte count (10^9^/L), and the absolute platelets count(10^9^/L) to the absolute lymphocyte count(10^9^/L), respectively; SII = platelet × NLR [19]; and SIRI = neutrophil × monocyte/lymphocyte counts [20]. The GPS and CONUT scores were estimated using the scoring systems described in Tables S1 and S2 (available in Supplementary Materials). The analysis also included other conventional factors, including demographic factors (age and sex), clinical factors (body mass index and comorbidity), tumor factors (TNM stage and number of metastatic sites), and laboratory factors (pretreatment and post-treatment EBV DNA levels). The calculated NRI, PNI, and other indicators were analyzed using Microsoft Excel (Redmond, WA, USA).

### 2.3. Treatments

All eligible patients received one of the following platinum-based chemotherapy regimens: (1) GP: gemcitabine (1000 mg/m^2^ intravenously on days 1 and 8 of a 21-day cycle) plus cisplatin (80–100 mg/m^2^ intravenously on day 1 of a 21-day cycle); (2) PF: cisplatin (80–100 mg/m^2^ intravenously on day 1 of a 21-day cycle) plus 5-fluorouracil (500 mg/m^2^ continuous intravenously infusion on days 1–5 of a 21-day cycle); (3) TP: paclitaxel (175–200 mg/m^2^ intravenously on day 1 of a 21-day cycle) or docetaxel (70–75 mg/m^2^ intravenously on day 1 of a 21-day cycle) plus cisplatin (75–80 mg/m^2^ intravenously on day 1 of a 21-day cycle); (4) TPF: paclitaxel (135 mg/m^2^ intravenously on day 1 of a 21-day cycle) or docetaxel (60 mg/m^2^ intravenously on day 1 of a 21-day cycle) plus cisplatin (60 mg/m^2^ intravenously on day 1 of a 21-day cycle) plus 5- fluorouracil (600 mg/m^2^, continuous intravenously infusion on days 1–5 of a 21-day cycle), or oral capecitabine (1000 mg/m^2^ orally twice daily, days 1–14 of a 21-day cycle) [21]. Patients received anti-PD-1 agents every 3 weeks, including camrelizumab (200 mg) [22], toripalimab (3 mg/kg) [23], sintilimab (200 mg) [24], tislelizumab (200 mg) [25], or nivolumab (3 mg/kg or 240 mg) [26], until unacceptable toxicity developed, the disease progressed, the patient withdrew informed consent, or the investigator deemed it necessary, up to a maximum of 2 years.

### 2.4. Endpoints and Follow-Up

The primary endpoint was progression-free survival (PFS), which was defined as the interval from the start date of chemotherapy combination PD-1 inhibitor to disease progression or death from any cause. The secondary endpoints included overall survival (OS), objective response rate (ORR), and disease control rate (DCR). OS duration was measured from the first day of treatment to death from any cause. Tumor response was assessed every two treatment cycles, starting with the initiation of chemotherapy combination PD-1 inhibitor until progression or the start of another anticancer treatment. Objective response (OR) was defined as complete response (CR) or partial response (PR) with at least one sequential tumor assessment confirmed, according to revised Response Evaluation Criteria in Solid Tumors (RECIST v1.1). Disease control (DC) was defined as radiologically confirmed CR, PR, and stable disease (SD). Patients were followed up at least every 3 months after treatment completion. Each follow-up visit assessment included a complete physical examination, nasopharyngoscopy, head and neck MRI, chest X-ray or computed tomography scan, abdominal sonography or computed tomography scan, bone scans or [^18^F] FDG PET–CT, and plasma EBV DNA assay.

### 2.5. Statistical Analysis

Categorical variables were compared using the chi-square test or Fisher’s exact test. All continuous variables were converted into binary based on the optimal cut-off point determined by maximally selected rank statistics. Cox proportional hazards models were used to assess the prognostic value of each candidate index and calculate the corresponding hazard ratio (HR). Covariates with a *p*-value < 0.1 in univariate analyses were included in subsequent backward stepwise multivariate analyses. The 6-month PFS has been recommended as an end-point for checkpoint-inhibitor monotherapy trials [27]. Consequently, we utilized 1-year and 18-month PFS as time points for constructing a nomogram to assess the effectiveness of combined immunochemotherapy. The nomogram model incorporated all significant factors identified in the multivariate analysis, as well as important prognostic factors. The calibration capacities were evaluated using a calibration plot. The predictive performance of the nomogram was evaluated using the area under the receiver operating characteristic (ROC) curve (AUC) and was compared with that of other traditional or constituent factors. Decision curve analysis (DCA) was conducted to estimate the clinical utility of this model. Bootstraps with 1000 re-samples and 10-fold cross-validation were applied to avoid overfitting. We then stratified the patients into two categories based on the cut-off value of the total points derived from the established nomogram: favorable- and unfavorable- prognosis. Kaplan–Meier curves were used to estimate the time-to-event data of the favorable- and unfavorable- prognosis groups and were compared using the log-rank test. All statistical analyses were performed using Jamovi 2.3.26, SPSS 24.0, or R 4.2.1 (R project, http://www.R-project.org/; accessed on 16 June 2022, version 4.2.1, R Core Team, Vienna, Austria). Statistical significance was defined as a two-sided *p* value of less than 0.05.

## 3. Results

### 3.1. Patient Characteristics and Treatment Outcomes

No significant differences existed between the training and validation cohorts in the baseline characteristics (Table 1). The patients included 110 men (84.0%) and 21 women (16.0%), with a median age of 46 years (interquartile range, 38–53 years).

As of the last follow-up date of 30 April 2023, the median patient follow-up time was 34.7 months (interquartile range, 23.8–58.3 months). Among the patients who received the chemotherapy combination PD-1 inhibitor, 84 (64.1%) patients experienced disease progression and 47 (35.9%) patients had died. The median progression-free survival (mPFS) of the entire cohort was 18.87 months (95% CI: 12.86–24.87 months) with 1-year, 2-year, and 3-year probabilities of PFS at 59.1%, 41.1%, and 25.9%, respectively. The objective response rate (ORR) was 72.5% (95% CI: 64.3–79.4%), and the disease control rate (DCR) was 84.0% (95% CI: 76.7–89.3%).

### 3.2. Factors Associated with Disease Progression

The optimal cut-off values determined by the maximally selected rank statistics in the whole dataset for BMI, NRI, PNI, SII, SIRI, LAR, LMR, NLR, PLR, and pretreatment and post-treatment EBV DNA were identified to be 19.19 kg/m^2^, 108.08, 49.20, 521.32, 2.42, 3.74, 2.87, 3.24, 123.0, 4760, and 99.50 copies/mL, respectively (Supplementary Materials). Univariate and multivariate Cox regression results are presented in Table 2. A total of 11 variables (*p* < 0.1) were selected for multivariate analysis from the univariate analysis of the training cohort. The stepwise multivariate analysis revealed that baseline UA level (HR: 5.026, 95% confidence interval (Cl): 1.532–16.497, *p* = 0.008), PNI (HR: 0.096, 95% Cl: 0.030–0.309, *p* < 0.001), NRI (HR: 0.176, 95% Cl: 0.066–0.464, *p* < 0.001), and post-treatment EBV DNA level (HR: 3.109, 95% CI: 1.502–6.437, *p* < 0.003) were identified as independent significant prognostic factors for PFS (Table S1). Notably, the SII showed a possible association with disease progression in the univariate analysis (HR: 2.088, 95% Cl: 0.987–4.418, *p* = 0.054), whereas it was not a significant predictor in the multivariate analysis (HR: 1.990, 95% Cl: 0.732–5.413, *p* = 0.177). Despite this, considering its potential prognostic value, the SII was incorporated into the final model as well.

### 3.3. Development of a Nomogram Model for PFS

**Figure 1:**
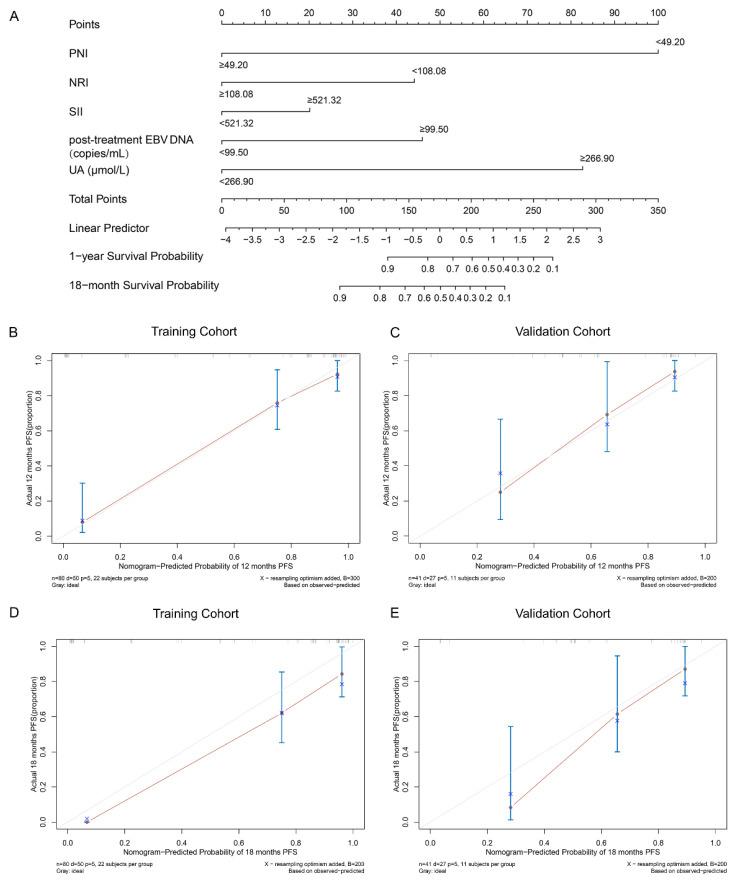
Nomogram (**A**) established based on nutritional indexes, inflammatory parameter, and post-treatment EBV DNA level for predicting 1-year and 18-month PFS in patients with dmNPC undergoing chemotherapy combination PD-1 inhibitor. Each variable was assigned a score based on its contribution to the outcome event. The total points for each patient can be calculated by summing the allocated scores for each factor in the nomogram. A higher total score was associated with a poorer prognosis. The calibration curves in the training cohort ((**B**,**D**), respectively) and validation ((**C**,**E**), respectively) cohorts. Abbreviations: NRI = nutritional risk index; PNI = prognostic nutritional index; SII = systemic immune-inflammation index; EBV DNA = Epstein–Barr virus DNA; UA = uric acid; PFS = progression-free survival.

A nomogram was developed to predict the PFS of patients with dmNPC who received first-line or subsequent-line chemotherapy combined with PD-1 inhibitor. This nomogram was constructed based on several prognostic factors, including PNI (<49.20 or ≥49.20), NRI (<108.08 or ≥108.08), SII (<521.32 or ≥521.32), UA (<266.90 or ≥266.90 μmol/L), and post-treatment EBV DNA level (<99.50 or ≥99.50 copies/mL) (Figure 1A). The calibration of the established nomogram for predicting 1-year and 18-month PFS demonstrated good agreement between the nomogram-based predictions and the observed outcomes in both the training and validation cohorts (Figure 1B).

**Figure 2:**
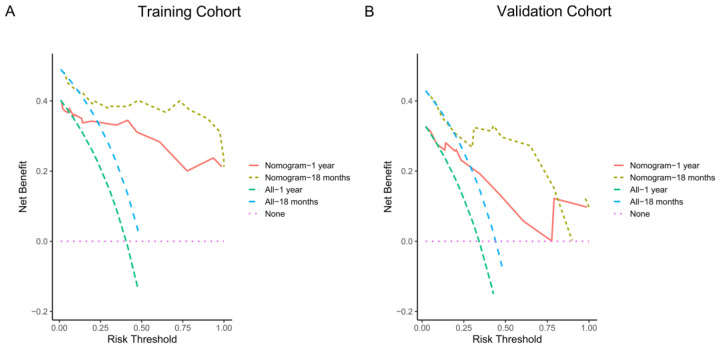
Decision curves analyses of the established nomogram predicting progression-free survival (PFS) at 1 year and 18 months in the training (**A**) and validation (**B**) cohort.

The DCA used to evaluate the potential clinical application of this nomogram in the training and validation cohorts is presented in Figure 2, which shows that the nomogram provided satisfactory performance.

### 3.4. Comparison and Validation of the Predictive Accuracy of Nomogram and Other Traditional Factors

**Figure 3:**
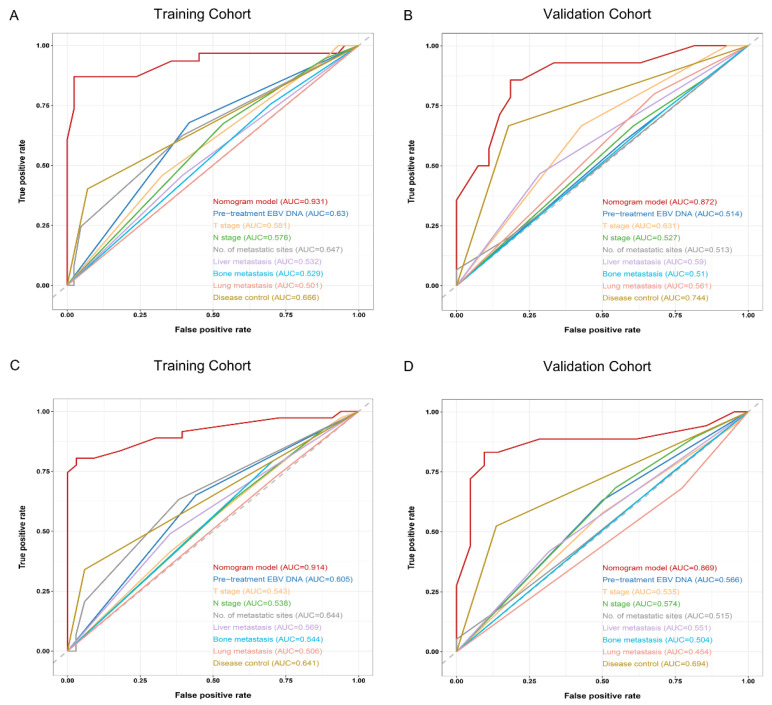
The predictive accuracy of the established nomogram against other traditional baseline factors for 1-year and 18-month PFS in the training ((**A**,**C**), respectively) and validation ((**B**,**D**), respectively) cohorts. Abbreviations: EBV DNA = Epstein–Barr virus DNA; PFS = progression-free survival.

The ROC curves clearly demonstrated that the nomogram exhibited superior discriminative ability in predicting 1-year and 18-month PFS compared to any of the traditional baseline factors, as observed in both the training and validation cohorts (Figure 3).

### 3.5. Comparison and Validation of the Predictive Accuracy of Nomogram and Other Constituent Factors

**Figure 4:**
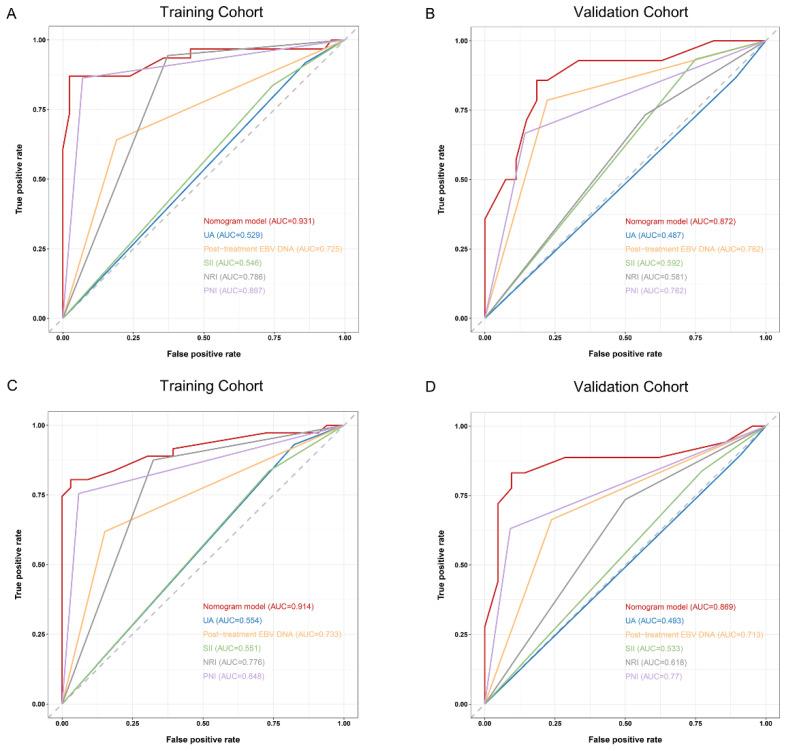
The predictive accuracy of the established nomogram against other constituent factors for 1-year and 18-month progression-free survival (PFS) in the training ((**A**,**C**), respectively) and validation ((**B**,**D**), respectively) cohorts.

The predictive efficiency for PFS in patients with dmNPC was compared among nomogram, PNI, NRI, SII, UA, and post-treatment EBV DNA. The AUC of the nomogram was also significantly superior to that of any independent factor, both in the training and validation cohorts (Figure 4).

### 3.6. Separating Patients into Different Risk Groups

**Figure 5:**
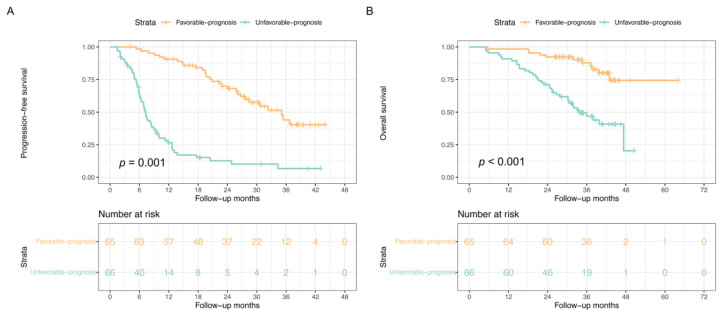
Survival curves of the favorable- and unfavorable-prognosis groups stratified by the nomogram for progression-free survival (**A**) and overall survival (**B**) in the whole cohort. Log-tank test was used to calculate the *p*-value.

According to the best cut-off values of the total score derived from the nomogram, all patients were categorized into two risk groups: a favorable-prognosis group (total scores < 164 points) and an unfavorable-prognosis group (total scores ≥ 164 points). Figure 5 illustrates the survival curves for PFS and OS of these prognostic groups. The favorable-prognosis group exhibited significantly longer survival outcomes compared to the unfavorable-prognosis group (mPFS, 35.10 months [95%CI:27.36–42.84] vs. 7.23 months [95%CI: 6.50–7.97], *p* = 0.001; mOS, not reached vs. 33.73 months [95%CI: 36.73–40.73], *p* < 0.001) (Table 3).

**Figure 6:**
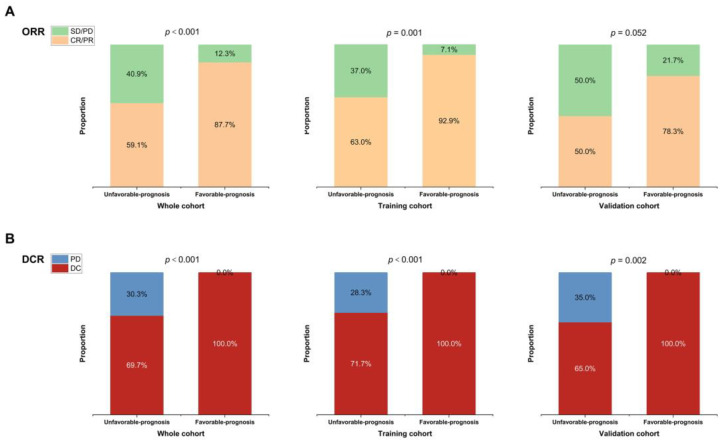
The distribution of ORR (**A**) and DCR (**B**) for the favorable- and unfavorable-prognosis groups in the whole cohort, training cohort, and validation cohort. Chi-square tests were performed to compare the distribution between different prognosis groups. Abbreviations: ORR = objective response rate; DCR = disease control rate; CR = complete response; PR = partial response; SD = stable disease; PD = progressive disease; DC = disease control.

In addition, we discovered that the ORR was higher in the favorable-prognosis group than in the unfavorable-prognosis group (pooled analysis, 87.7% [95% CI: 77.6–93.6%] vs. 59.1% [95% CI: 47.1–70.1%], *p* < 0.001; training cohort: 92.9% [95% CI: 81.0–40.0%] vs. 63.0% [95% CI: 48.6–75.5%], *p* = 0.001; validation cohort: 78.3% [95% CI: 58.1–90.3%] vs. 50.0% [95% CI: 30.0–70.1%], *p* = 0.052; Figure 6A). Additionally, the DCR was better in the favorable-prognosis group than in the unfavorable-prognosis group (pooled analysis, 100.0% [95% CI: 94.4–100.0%] vs. 69.7% [95% CI: 57.8–79.5%], *p* < 0.001; training cohort, 100.0% [95% CI: 91.6–100.0%] vs. 71.7% [95% CI: 57.5–82.7%], *p* < 0.001; validation cohort, 100.0% [95% CI: 85.7–100.0%] vs. 65.0% [95% CI: 43.3–81.9%], *p* = 0.002, Figure 6B).

## 4. Discussion

This study established and validated a combined model that integrates nutritional indexes, inflammatory parameters, EBV DNA, and biochemistry profiling to predict survival without disease progression in patients with dmNPC undergoing chemotherapy combination PD-1 inhibitor treatment. According to the nomogram scores, we developed a risk stratification system that could allocate patients into favorable- and unfavorable-prognosis groups. Furthermore, this prognostic model serves as a clinically useful tool for individualized survival prediction and aids in formulating personalized surveillance recommendations for dmNPC.

The development of NPC is strongly associated with EB virus infection in epidemic areas [28]. Therefore, there is abundant lymphocyte infiltration and high-level programmed death ligand-1 (PD-L1) expression in the tumor region, which makes immunotherapy a promising choice for the treatment of NPC [29]. Recently, platinum-based chemotherapy in combination with PD-1 inhibitor has been recommended as the primary treatment option for dmNPC [30]. However, the outcomes of patients at the same stage receiving the same treatment may be completely different. Moreover, there is currently no standard follow-up strategy for patients with dmNPC following combined chemoimmunotherapy treatment; treatment options beyond the first line of therapy are limited [31]. Thus, it is imperative to identify alternative biomarkers that can predict treatment outcomes.

Malnutrition has been proven to have adverse effects on the body’s immune system, therapeutic efficacy, and tolerance to interventions, thereby exacerbating disease progression, local recurrence, and distant metastasis [32]. Malnutrition has been reported to occur in 35–60% of patients with NPC, significantly impacting treatment responses [33]. However, traditional nutritional parameters such as body mass index (BMI) and serum albumin (ALB) have certain limitations in estimating the nutritional status of cancer patients. For example, BMI may not accurately reflect changes in body fat and muscle mass proportions with age [34]. In this study, we verified that the nutritional risk index (NRI), which is calculated based on the patient’s height, weight, and serum albumin level, provided more reliable predictive power for survival outcomes in patients with dmNPC compared to body mass index (BMI) and albumin alone. Our findings indicated that patients in the high NRI score group exhibited better PFS than those in the low NRI score group, which is in agreement with the results of a previous study investigating single nutritional parameter in NPC [35]. Regarding another nutritional parameter related to the PFS of patients with dmNPC in our study, the prognostic nutritional index (PNI) was also reported as a prognostic factor for the distant metastasis-free survival (DMFS), disease-specific survival (DSS) and overall survival (OS) in patients with NPC treated with intensity-modulated radiotherapy (IMRT) [6]. In addition to serum albumin, PNI also incorporates the lymphocyte count in the peripheral blood, which is a crucial biomarker of the host’s cellular adaptive immune response against cancer cells [36]. Therefore, PNI is widely regarded as a reliable indicator that reflects both the nutritional and immune status of patients [37].

On the other hand, inflammation associated with cancer is acknowledged as a cancer hallmark that affects all stages of malignancies, including tumorigenesis, proliferation, invasion, and metastasis [38]. In our study, high SII score was related to worse PFS in univariate Cox regression analyses (*p* = 0.054; HR, 2.088; 95% CI, 0.987–4.418); however, multivariate analyses revealed no significant impact of SII (*p* = 0.177; HR, 1.990; 95% CI, 0.732–5.413) in predicting PFS. Considering the correlation between SII scores and tumor progression, we also incorporated it into the final model. The underlying biological mechanism linking high inflammatory parameters to a poor prognosis in cancer patients remains controversial. The prognostic value of SII may be explained by the roles of its components. Firstly, circulating neutrophils secrete large amounts of arginase, nitric oxide, and ROS, which can interfere with T-cell activation [39]. Secondly, lymphocytes could inhibit the proliferation and metastasis of cancer cells and then affect the host immune response [40]. Lastly, platelets play a role in protecting circulating tumor cells (CTCs) from shear stresses in the circulation, inducing epithelial-to-mesenchymal transition (EMT) of CTCs and promoting their infiltration into metastatic sites [41].

In this study, it was demonstrated that post-treatment EBV- DNA, rather than pre-treatment EBV- DNA, was a prognostic factor that significantly correlated with the outcomes of patients receiving combined immunochemotherapy. This finding was consistent with a previous prospective multicenter study [42]. Most early events in NPC are caused by disease progression or recurrence, which can be predicted by post-treatment EBV- DNA. This is because post-treatment EBV- DNA may reflect minimal residual disease at the end of treatment. As for the biochemical indicators, both univariate and multivariate analyses indicated that a high baseline serum uric acid (UA) level (>266.90 μmol/L) was an unfavorable prognostic factor for dmNPC, which is in accordance with the research of Du et al. [43]. They reported that serum UA level (>353.4 μmol/L) was an adverse feature for patients with locally advanced NPC. Uric acid, an antioxidant, plays a crucial role in protecting against DNA damage, weakening cell migration ability, eliminating reactive oxygen free radicals, and regulating cell death [44]. High serum UA levels may represent tumor burden, as the rapid proliferation and destruction of tumor cells lead to increased nucleic acid turnover [45].

Nevertheless, the capacity of a single parameter to evaluate the benefit of combined chemoimmunotherapy remains limited. Therefore, we integrated the aforementioned five independent prognostic factors to construct a nomogram model that can predict the probability of PFS after chemotherapy combination PD-1 inhibitor treatment in individual dmNPC patients. The combination of these five risk factors showed superior predictive effeciency than the individual factor. Based on this model, we stratified patients with dmNPC into favorable- or unfavorable-prognosis groups. Patients with a favorable prognosis were more likely to benefit from chemotherapy combined with PD-1 inhibitor treatment, while those with an unfavorable prognosis may progress sooner. Hence, such unfavorable-prognosis patients might require additional intensive therapeutic interventions, such as a combination of anti-VEGF therapies, anti-EGFR therapies, and locoregional radiotherapy.

However, this study also has some limitations. Firstly, the presence of certain selection biases are unavoidable due to the retrospective nature of the study. Secondly, the most common type of NPC in epidemic areas is associated with EBV infection, which may exhibit different tumor characteristics compared to low-risk areas. Finally, our study only enrolled patients from a single center and lacked external validation. Thus, the power of this prognostic model should be further validated in prospective clinical studies with multi-center cohorts.

## 5. Conclusions

In conclusion, our study proposed a combination of baseline nutritional and inflammatory indicators, post-treatment EBV DNA level, and laboratory examinations in a nomogram as possible prognostic biomarkers to predict 1-year and 18-month PFS for patients with dmNPC receiving combined immunochemotherapy. The proposed risk model had significantly better discrimination over other traditional indicators. Thus, it may be a useful tool for individualized assessment of the prognosis and personalized surveillance for patients with dmNPC treated with immunotherapy and chemotherapy.

## Figures and Tables

**Table 1:** Patient demographics and clinical characteristics of the whole cohort *.

Characteristic	Training Cohort (*n* = 88) No. (%)	Validation Cohort (*n* = 43) No. (%)	*p* Value
Sex			0.842
Female	15 (17%)	6 (14%)	
Male	73 (83%)	37 (86%)	
Smoking			0.362
No	57 (64.8%)	32 (74.4%)	
Yes	31 (35.2%)	11 (25.6%)	
Drinking			0.480
No	72 (81.8%)	38 (88.4%)	
Yes	16 (18.2%)	5 (11.6%)	
Family history			0.394
No	85 (96.6%)	40 (93%)	
Yes	3 (3.4%)	3 (7%)	
Age	45.85 ± 11.14	47.63 ± 11.50	0.404
Height (cm)	165.85 ± 6.17	164.17 ± 8.09	0.233
Weight (kg)	63.30 ± 10.50	61.49 ± 11.58	0.174
^a^ Tumor stage			0.487
T1	3 (3.4%)	0 (0%)	
T2	4 (4.5%)	2 (4.7%)	
T3	45 (51.1%)	19 (44.2%)	
T4	36 (40.9%)	22 (51.2%)	
^a^ Node stage			0.488
N1	8 (9.1%)	6 (14.0%)	
N2	28 (31.8%)	10 (23.3%)	
N3	52 (59.1%)	27 (62.8%)	
Pretreatment EBV DNA, copies/mL			0.527
<4760	42 (47.7%)	18 (41.9%)	
≥4760	46 (52.3%)	25 (58.1%)	
Post-treatment EBV DNA, copies/mL			0.821
<99.50	50 (62.5%)	24 (58.5%)	
≥99.50	30 (37.5%)	17 (41.5%)	
Liver metastasis			0.472
No	50 (56.8%)	28 (65.1%)	
Yes	38 (43.2%)	15 (34.9%)	
Bone metastasis			0.952
No	25 (28.4%)	12 (27.9%)	
Yes	63 (71.6%)	31 (72.1%)	
Lung metastasis			0.799
No	60 (68.2%)	31 (72.1%)	
Yes	28 (31.8%)	12 (27.9%)	
Distance LN metastasis			0.939
No	64 (72.7%)	31 (72.1%)	
Yes	24 (27.3%)	12 (27.9%)	
No of metastatic sites			0.333
1	42 (47.7%)	26 (60.5%)	
2–3	41 (46.6%)	16 (37.2%)	
≥4	5 (5.7%)	1 (2.3%)	
Chemotherapy combination PD-1 inhibitor lines			0.363
1	66 (75.0%)	29 (67.4%)	
≥2	22 (25.0%)	14 (32.6%)	
Response			0.233
CR	2 (2.3%)	0 (0%)	
PR	66 (75.0%)	28 (65.1%)	
SD	7 (8.0%)	8 (18.6%)	
PD	13 (14.8%)	7 (16.3%)	
Anti-PD-1 agent			0.651
Camrelizumab	27 (30.7%)	14 (32.6%)	
Toripalimab	44 (50.0%)	18 (41.9%)	
Sintilimab	7 (8.0%)	7 (16.3%)	
Tislelizumab	8 (9.1%)	3 (7.0%)	
Nivolumab	2 (2.3%)	1 (2.3%)	
Chemotherapy regimens			0.470
GP	60 (68.2%)	23 (53.5%)	
PF	3 (3.4%)	3 (7.0%)	
TP	10 (11.4%)	6 (14.0%)	
Capecitabine	4 (4.6%)	4 (9.3%)	
TPF	1 (1.1%)	2 (4.7%)	
Others	10 (11.4%)	5 (11.6%)	
BMI (kg/m^2^)			0.816
<19.19	11 (12.5%)	6 (14%)	
≥19.19	77 (87.5%)	37 (86%)	
NRI			0.876
<108.08	58 (65.9%)	27 (62.8%)	
≥108.08	30 (34.1%)	16 (37.2%)	
PNI			0.223
<49.20	40 (45.5%)	14 (32.6%)	
≥49.20	48 (54.5%)	29 (67.4%)	
SII			0.754
<521.32	20 (22.7%)	8 (18.6%)	
≥521.32	68 (77.3%)	35 (81.4%)	
SIRI			0.447
<2.42	74 (84.1%)	39 (90.7%)	
≥2.42	14 (15.9%)	4 (9.3%)	
GPS			
0	58 (65.9%)	34 (79.1%)	
1–2	30 (34.1%)	9 (20.9%)	
CONUT score			
0–1	50 (56.8%)	25 (58.1%)	
2–6	38 (43.2%)	18 (41.9%)	
NLR			0.608
<3.24	58 (65.9%)	31 (72.1%)	
≥3.24	30 (34.1%)	12 (27.9%)	
LAR			0.359
<3.74	15 (17%)	11 (25.6%)	
≥3.74	73 (83%)	32 (74.4%)	
LMR			0.443
<2.87	21 (23.9%)	7 (16.3%)	
≥2.87	67 (76.1%)	36 (83.7%)	
PLR			0.999
<123.0	22 (25%)	10 (23.3%)	
≥123.0	66 (75%)	33 (76.7%)	
WBC (109/L)			0.652
<9.18	72 (81.8%)	33 (76.7%)	
≥9.18	16 (18.2%)	10 (23.3%)	
Neutrophil (109/L)			0.390
<7.26	78 (88.6%)	35 (81.4%)	
≥7.26	10 (11.4%)	8 (18.6%)	
Lymphocyte (109/L)			0.727
<2.03	53 (60.2%)	28 (65.1%)	
≥2.03	35 (39.8%)	15 (34.9%)	
Monocyte (109/L)			0.987
<0.55	61 (69.3%)	29 (67.4%)	
≥0.55	27 (30.7%)	14 (32.6%)	
RBC (1012/L)			0.987
<4.49	18 (20.5%)	8 (18.6%)	
≥4.49	70 (79.5%)	35 (81.4%)	
PLT (109/L)			0.340
<374.0	75 (85.2%)	33 (76.7%)	
≥374.0	13 (14.8%)	10 (23.3%)	
HGB (g/L)			0.421
<145.0	47 (53.4%)	19 (44.2%)	
≥145.0	41 (46.6%)	24 (55.8%)	
ALP (U/L)			0.074
<94.10	73 (83%)	29 (67.4%)	
≥94.10	15 (17%)	14 (32.6%)	
GGT (U/L)			0.356
<32.80	54 (61.4%)	22 (51.2%)	
≥32.80	34 (38.6%)	21 (48.8%)	
LDH (U/L)			0.231
<163.30	10 (11.4%)	9 (20.9%)	
≥163.30	78 (88.6%)	34 (79.1%)	
UA (μmol/L)			0.825
<266.90	13 (14.8%)	5 (11.6%)	
≥266.90	75 (85.2%)	38 (88.4%)	
GLU (mmol/L)			0.921
<4.97	34 (38.6%)	17 (39.5%)	
≥4.97	54 (61.4%)	26 (60.5%)	
CRP (mg/L)			0.772
<27.0	77 (87.5%)	39 (90.7%)	
≥27.0	11 (12.5%)	4 (9.3%)	
CK (U/L)			0.822
<47.0	13 (14.8%)	7 (16.3%)	
≥47.0	75 (85.2%)	36 (83.7%)	
SAA (mg/L)			0.444
<19.30	56 (63.6%)	31 (72.1%)	
≥19.30	32 (36.4%)	12 (27.9%)	

* *p*-value was conducted with the chi-square test (categorical variables) and Mann–Whitney U test (continuous variables), respectively. Abbreviations: EBV DNA, Epstein–Barr virus DNA; LN, lymph nodes; PD-1, programmed death-1; CR, complete response; PR, partial response; SD, stable disease; PD, progressive disease; BMI, body mass index; NRI, nutritional risk index; PNI, prognostic nutritional index; SII, systemic immune-inflammation index; SIRI, systemic inflammatory response index; GPS, Glasgow prognostic score; CONUT, controlling nutritional status; NLR, neutrophil-to-lymphocyte ratio; LAR, lactate dehydrogenase-to-albumin ratio; LMR, lymphocyte-to-monocyte ratio; PLR, platelet-to-lymphocyte ratio; WBC, white blood cell; RBC, red blood cell; HGB, hemoglobin; ALP, alkaline phosphatase; GGT, gamma-glutamyl transferase; LDH, lactate dehydrogenase; UA, uric acid; GLU, glucose; CRP, C-reactive protein; CK, creatine kinase; SAA, serum amyloid A. ^a^ According to the eighth edition of UICC/AJCC staging system.

**Table 2:** Univariate and multivariable analysis of the training cohort *.

Variables	Univariate HR (95%CI)	*p* Value	Multivariate HR (95%CI)	*p* Value
Sex		0.555		
Female	Reference			
Male	1.254 (0.592, 2.654)			
Smoking		0.942		
No	Reference			
Yes	1.020 (0.593, 1.755)			
Drinking		0.247		
No	Reference			
Yes	1.451 (0.773, 2.722)			
Family history		0.665		
No	Reference			
Yes	1.369 (0.330, 5.675)			
Age	1.011 (0.987, 1.036)	0.369		
^a^ Tumor stage		0.620		
T1	Reference			
T2	1.501 (0.136, 16.601)	0.740		
T3	2.704 (0.368, 19.878)	0.328		
T4	2.888 (0.389, 21.413)	0.300		
^a^ Node stage		0.543		
N1	Reference			
N2	1.808 (0.618, 5.296)	0.280		
N3	1.747 (0.616, 4.954)	0.294		
Pretreatment EBV DNA, copies/mL		0.128		
<4760	Reference			
≥4760	1.516 (0.887, 2.592)			
Post-treatment EBV DNA, copies/mL		<0.001		0.003
<99.50	Reference		Reference	
≥99.50	0.387 (0.253, 0.592)		3.109 (1.502, 6.437)	
Liver metastasis		0.420		
No	Reference			
Yes	1.243 (0.733, 2.106)			
Bone metastasis		0.181		
No	Reference			
Yes	1.534 (0.820, 2.871)			
Lung metastasis		0.342		
No	Reference			
Yes	0.750 (0.415, 1.356)			
Distance LN metastasis		0.146		
No	Reference			
Yes	1.535 (0.861, 2.736)			
No of metastatic sites		0.175		
1	Reference			
2–3	2.200 (0.871, 5.559)	0.096		
≥4	2.884 (0.833, 9.987)	0.095		
Chemotherapy combination PD-1 inhibitor lines		0.395		
1	Reference			
≥2	1.339 (0.683, 2.626)			
Response		<0.001		0.791
CR	Reference		Reference	
PR	2.325 (0.318, 17.004)	0.406	2.159 (0.242, 19.237)	0.490
SD	2.489 (0.253, 24.469)	0.434	1.651 (0.125, 21.738)	0.703
PD	17.029 (2.108, 137.588)	0.008	1.558 (0.139, 17.476)	0.719
Anti-PD-1 agent		0.923		
Camrelizumab	Reference			
Toripalimab	0.958 (0.530, 1.733)	0.888		
Sintilimab	0.704 (2.108, 137.588)	0.574		
Tislelizumab	17.029 (2.108, 137.588)	0.682		
Nivolumab	17.029 (2.108, 137.588)	0.573		
Chemotherapy regimens		0.005		0.197
GP	Reference		Reference	
PF	0.290 (0.040, 2.134)	0.224	0.100 (0.012, 0.805)	0.030
TP	0.742 (0.265, 2.081)	0.571	0.526 (0.172, 1.603)	0.258
Capecitabine	0.816 (0.195, 3.411)	0.780	0.954 (0.168, 5.434)	0.958
TPF	13.237 (1.608, 108.983)	0.016	0 (0, -)	0.983
Others	3.121 (1.467, 6.637)	0.003	0.427 (0.147, 1.242)	0.118
BMI (kg/m^2^)		0.064		0.291
<19.19	Reference		Reference	
≥19.19	0.507 (0.248, 1.040)		1.849 (0.590, 5.791)	
NRI		<0.001		<0.001
<108.08	Reference		Reference	
≥108.08	0.201 (0.104, 0.389)		0.176 (0.066, 0.464)	
PNI		<0.001		<0.001
<49.20	Reference		Reference	
≥49.20	0.092 (0.047, 0.180)		0.096 (0.030, 0.309)	
SII		0.054		0.177
<521.32	Reference		Reference	
≥521.32	2.088 (0.987, 4.418)		1.990 (0.732, 5.413)	
SIRI		0.583		
<2.42	Reference			
≥2.42	0.810 (0.383, 1.717)			
GPS		0.875		
0	Reference			
1–2	0.958 (0.563, 1.631)			
CONUT score		0.222		
0–1	Reference			
2–6	1.130 (0.928, 1.376)			
NLR		0.130		
<3.24	Reference			
≥3.24	1.824 (1.058, 3.143)			
LAR		0.289		
<3.74	Reference			
≥3.74	1.536 (0.695, 3.395)			
LMR		0.276		
<2.87	Reference			
≥2.87	1.444 (0.746, 2.794)			
PLR		0.088		0.754
<123.0	Reference		Reference	
≥123.0	1.815 (0.915, 3.601)		1.154 (0.472, 2.821)	
WBC (109/L)		0.093		0.199
<9.18	Reference		Reference	
≥9.18	0.506 (0.228, 1.120)		0.439 (0.125, 1.542)	
Neutrophil (109/L)		0.559		
<7.26	Reference			
≥7.26	0.759 (0.301, 1.913)			
Lymphocyte (109/L)		0.168		
<2.03	Reference			
≥2.03	0.682 (0.396, 1.175)			
Monocyte (109/L)		0.297		
<0.55	Reference			
≥0.55	0.734 (0.411, 1.312)			
RBC (1012/L)		0.143		
<4.49	Reference			
≥4.49	0.604 (0.308, 1.186)			
PLT (109/L)		0.386		
<374.0	Reference			
≥374.0	0.703 (0.317, 1.558)			
HGB (g/L)		0.639		
<145.0	Reference			
≥145.0	0.882 (0.521, 1.492)			
ALP (U/L)		0.282		
<94.10	Reference			
≥94.10	0.662 (0.312, 1.404)			
GGT (U/L)		0.911		
<32.80	Reference			
≥32.80	0.970 (0.568, 1.655)			
LDH (U/L)		0.530		
<163.30	Reference			
≥163.30	1.344 (0.534, 3.382)			
UA (μmol/L)		0.077		0.008
<266.90	Reference		Reference	
≥266.90	2.513 (0.906, 6.969)		5.026 (1.532, 16.497)	
GLU (mmol/L)		0.007		0.393
<4.97	Reference		Reference	
≥4.97	0.479 (0.279, 0.821)		0.763 (0.411, 1.419)	
CRP (mg/L)		0.587		
<27.0	Reference			
≥27.0	0.791 (0.339, 1.846)			
CK (U/L)		0.373		
<47.0	Reference			
≥47.0	1.435 (0.649, 3.177)			
SAA (mg/L)		0.380		
<19.30	Reference			
≥19.30	0.780 (0.448, 1.358)			

* Hazard ratios estimated by Cox proportional hazard model. All variables were transformed into categorical variables. Abbreviations: HR, hazard ratio; CI, confidence interval; EBV DNA, Epstein–Barr virus DNA; LN, lymph nodes; PD-1, programmed death-1; CR, complete response; PR, partial response; SD, stable disease; PD, progressive disease; BMI, body mass index; NRI, nutritional risk index; PNI, prognostic nutritional index; SII, systemic immune-inflammation index; SIRI, systemic inflammatory response index; GPS, Glasgow prognostic score; CONUT, controlling nutritional status; NLR, neutrophil-to-lymphocyte ratio; LAR, lactate dehydrogenase-to-albumin ratio; LMR, lymphocyte-to-monocyte ratio; PLR, platelet-to-lymphocyte ratio; WBC, white blood cell; RBC, red blood cell; HGB, hemoglobin; ALP, alkaline phosphatase; GGT, gamma-glutamyl transferase; LDH, lactate dehydrogenase; UA, uric acid; GLU, glucose; CRP, C-reactive protein; CK, creatine kinase; SAA, serum amyloid A. ^a^ According to the eighth edition of UICC/AJCC staging system.

**Table 3:** Comparison of the survival of patients in different prognosis groups *.

Variable	PFS (Month)		OS (Month)	
	Median (95% CI)	*p*	Median (95% CI)	*p*
Favorable- prognosis	35.10 (27.36, 42.84)	0.001	- (-,-)	<0.001
Unfavorable- prognosis	7.23 (6.50, 7.97)		33.73 (36.73, 40.73)	

* *p*-value estimated by log-rank test. All statistical tests were two-sided. Abbreviations: CI, confidence interval; PFS, progression-free survival; OS, overall survival.

## Data Availability

The datasets generated during and/or analysed during the current study are available from the corresponding author on reasonable request.

## Supplementary Materials

The following supporting information can be downloaded at: https://www.mdpi.com/article/10.3390/nu15194262/s1, Figure S1. Flowchart showing the study design and patient selection process. Figure S2. Cutoff of body mass index (BMI) determined by maximally selected rank statistics and survival curves stratified by the cutoff. Figure S3. Cutoff of nutritional risk index (NRI) determined by maximally selected rank statistics and survival curves stratified by the cutoff. Figure S4. Cutoff of prognostic nutritional index (PNI) determined by maximally selected rank statistics and survival curves stratified by the cutoff. Figure S5. Cutoff of systemic immune-inflammation index (SII) determined by maximally selected rank statistics and survival curves stratified by the cutoff. Figure S6. Cutoff of systemic inflammatory response index (SIRI) determined by maximally selected rank statistics and survival curves stratified by the cutoff. Figure S7. Cutoff of lactate dehydrogenase-to-albumin ratio (LAR) determined by maximally selected rank statistics and survival curves stratified by the cutoff. Figure S8. Cutoff of lymphocyte-to-monocyte ratio (LMR) determined by maximally selected rank statistics and survival curves stratified by the cutoff. Figure S9. Cutoff of neutrophil-to-lymphocyte ratio (NLR) determined by maximally selected rank statistics and survival curves stratified by the cutoff. Figure S10. Cutoff of platelet-to-lymphocyte ratio (PLR) determined by maximally selected rank statistics and survival curves stratified by the cutoff. Figure S11. Cutoff of pretreatment Epstein–Barr virus DNA (EBV DNA) determined by maximally selected rank statistics and survival curves stratified by the cutoff. Figure S12. Cutoff of post-treatment Epstein–Barr virus DNA (EBV DNA) determined by maximally selected rank statistics and survival curves stratified by the cutoff. Table S1. Screening tool for Glasgow prognostic score. Table S2. Screening tool for controlling nutritional status score.

## Abbreviations

De novo metastatic nasopharyngeal carcinoma (dmNPC), programmed death-1(PD-1), nutritional risk index (NRI), prognostic nutritional index (PNI), Epstein–Barr virus DNA (EBV DNA), systemic immune-inflammation index (SII), uric acid (UA), progression-free survival (PFS), overall survival (OS).
