# Supplementary material for: Integrating Baseline Nutritional and Inflammatory Parameters with Post-Treatment EBV DNA Level to Predict Outcomes of Patients with De Novo Metastatic Nasopharyngeal Carcinoma Receiving Chemotherapy Combination PD-1 Inhibitor
Source: Nutrients. 2023 Oct 5;15(19):4262. doi: 10.3390/nu15194262 (PMC10574520; doi:10.3390/nu15194262)
Supplement: Supplementary file 1 [file nutrients-15-04262-s001.zip › nutrients-2620729-supplementary.pdf]

Figure S1. Flowchart showing the study design and patient selection process.

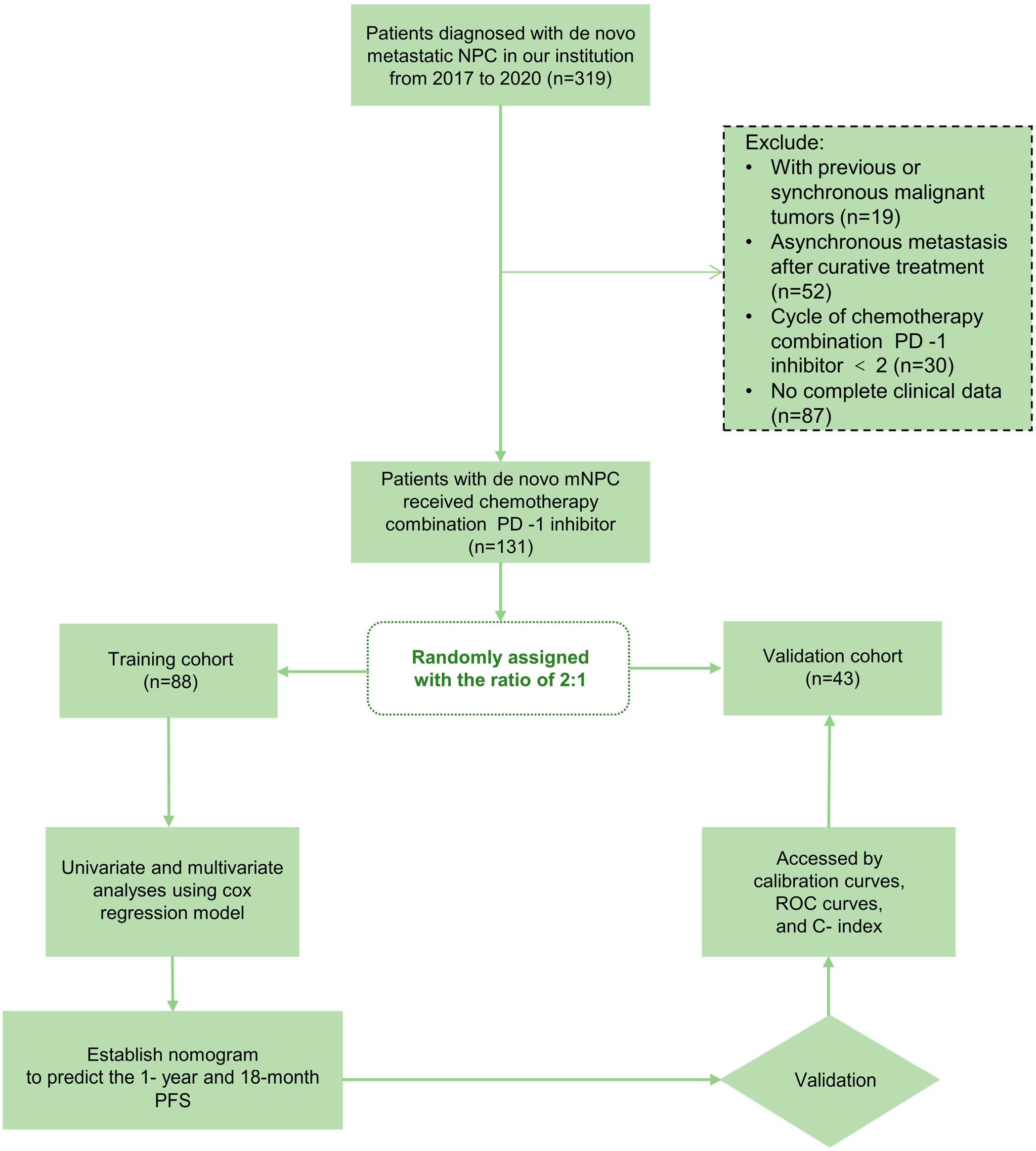

Abbreviations: mNPC, metastatic nasopharyngeal carcinoma; PD-1, programmed death-1; PFS, progression-free survival; ROC, receiver operating characteristic.

Figure S2. Cutoff of body mass index (BMI) determined by Maximally Selected Rank Statistics, and survival curves stratified by the cutoff.

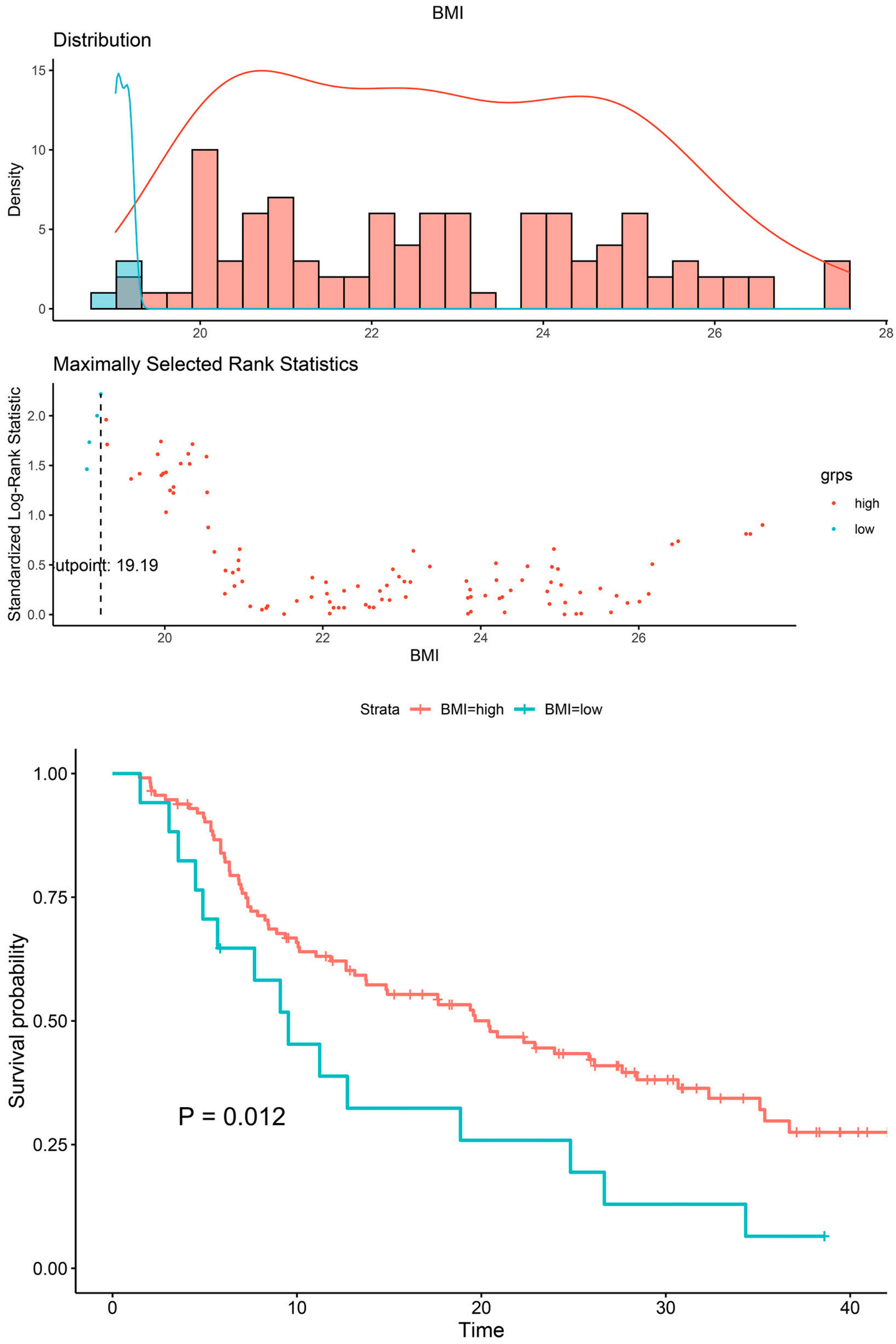

Figure S3. Cutoff of nutritional risk index (NRI) determined by Maximally Selected Rank Statistics, and survival curves stratified by the cutoff.

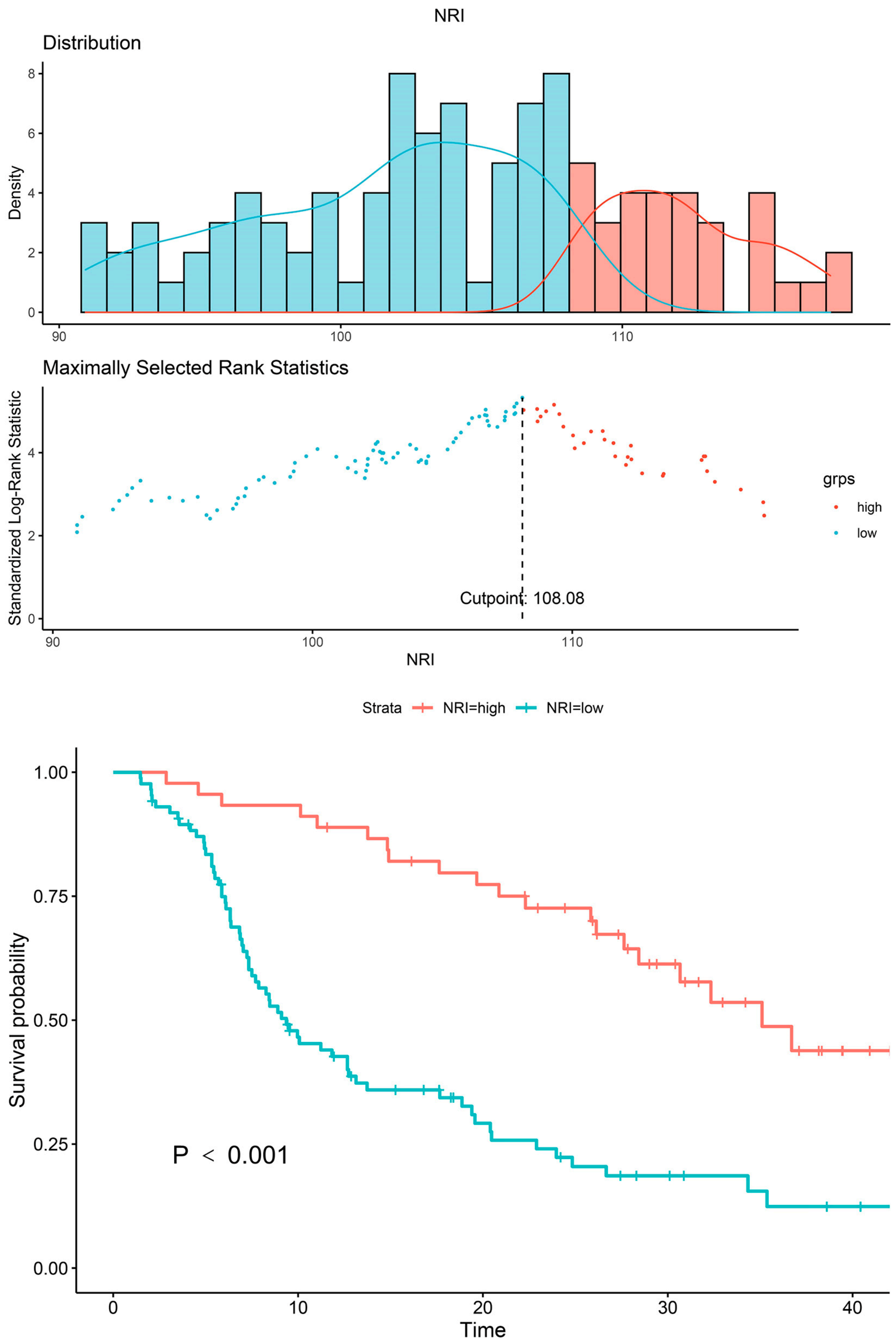

Figure S4. Cutoff of prognostic nutritional index (PNI) determined by Maximally Selected Rank Statistics, and survival curves stratified by the cutoff.

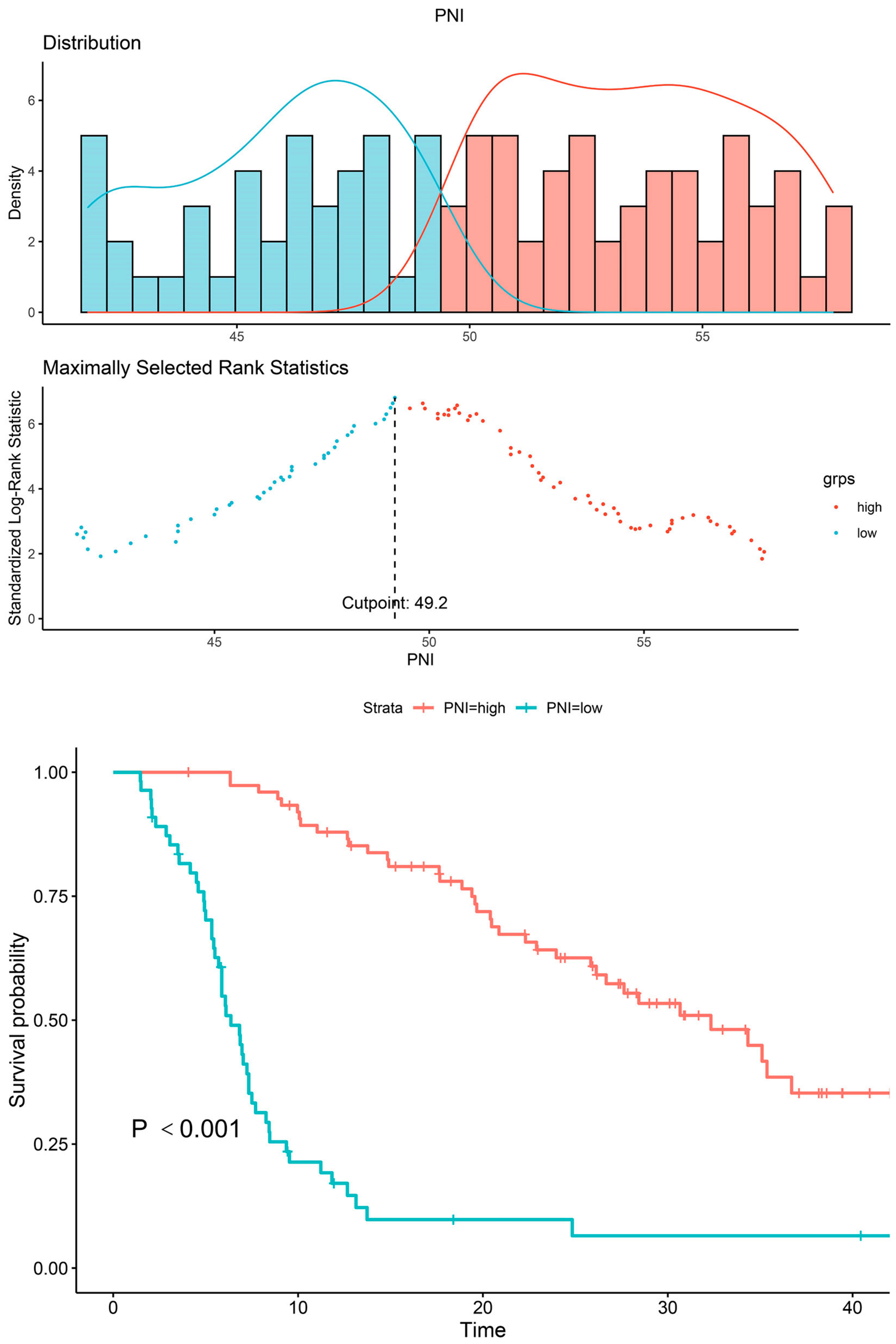

Figure S5. Cutoff of systemic immune-inflammation index (SII) determined by Maximally Selected Rank Statistics, and survival curves stratified by the cutoff.

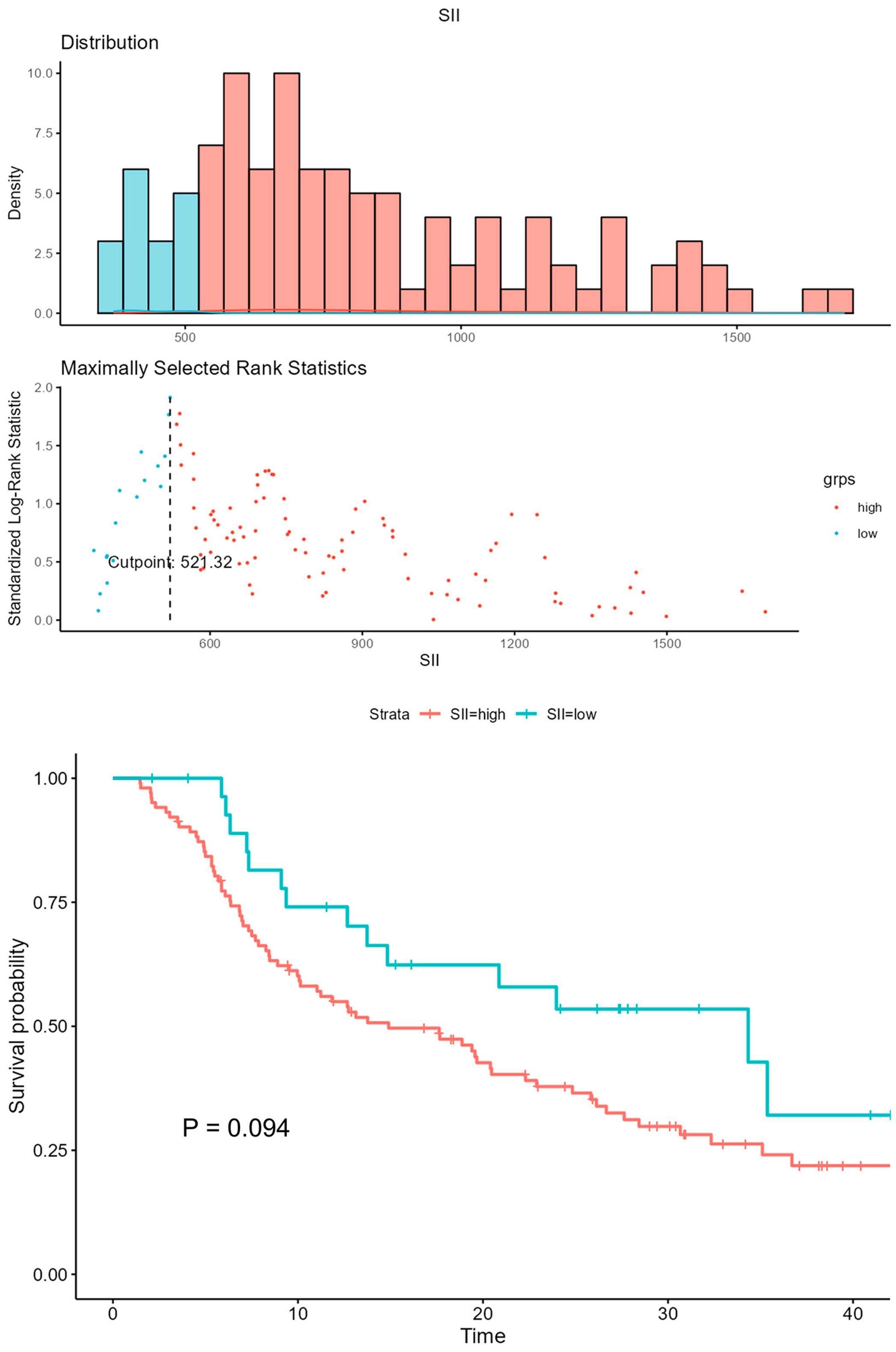

Figure S6. Cutoff of systemic inflammatory response index (SIRI) determined by Maximally Selected Rank Statistics, and survival curves stratified by the cutoff.

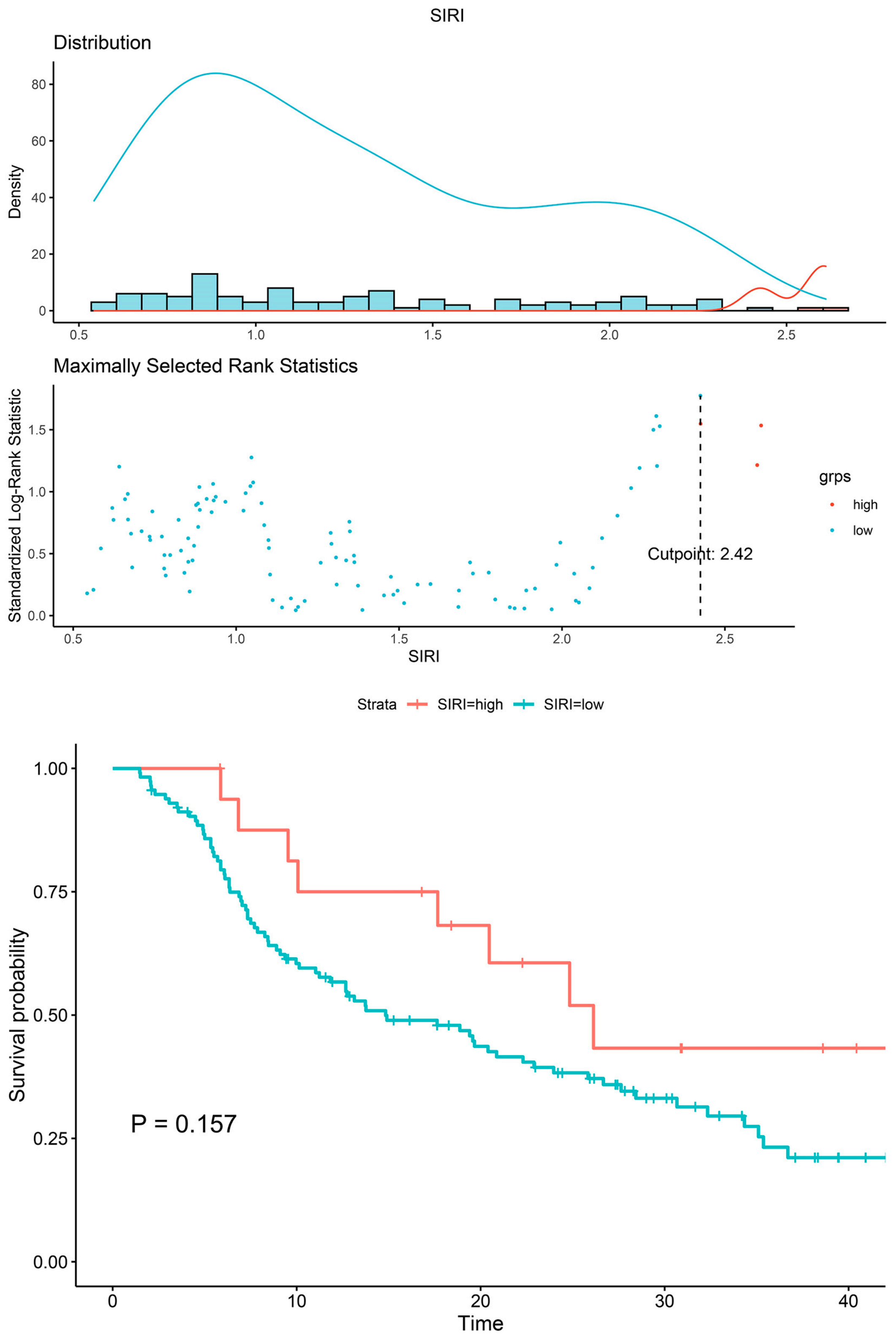

Figure S7. Cutoff of lactate dehydrogenase-to-albumin ratio (LAR) determined by Maximally Selected Rank Statistics, and survival curves stratified by the cutoff.

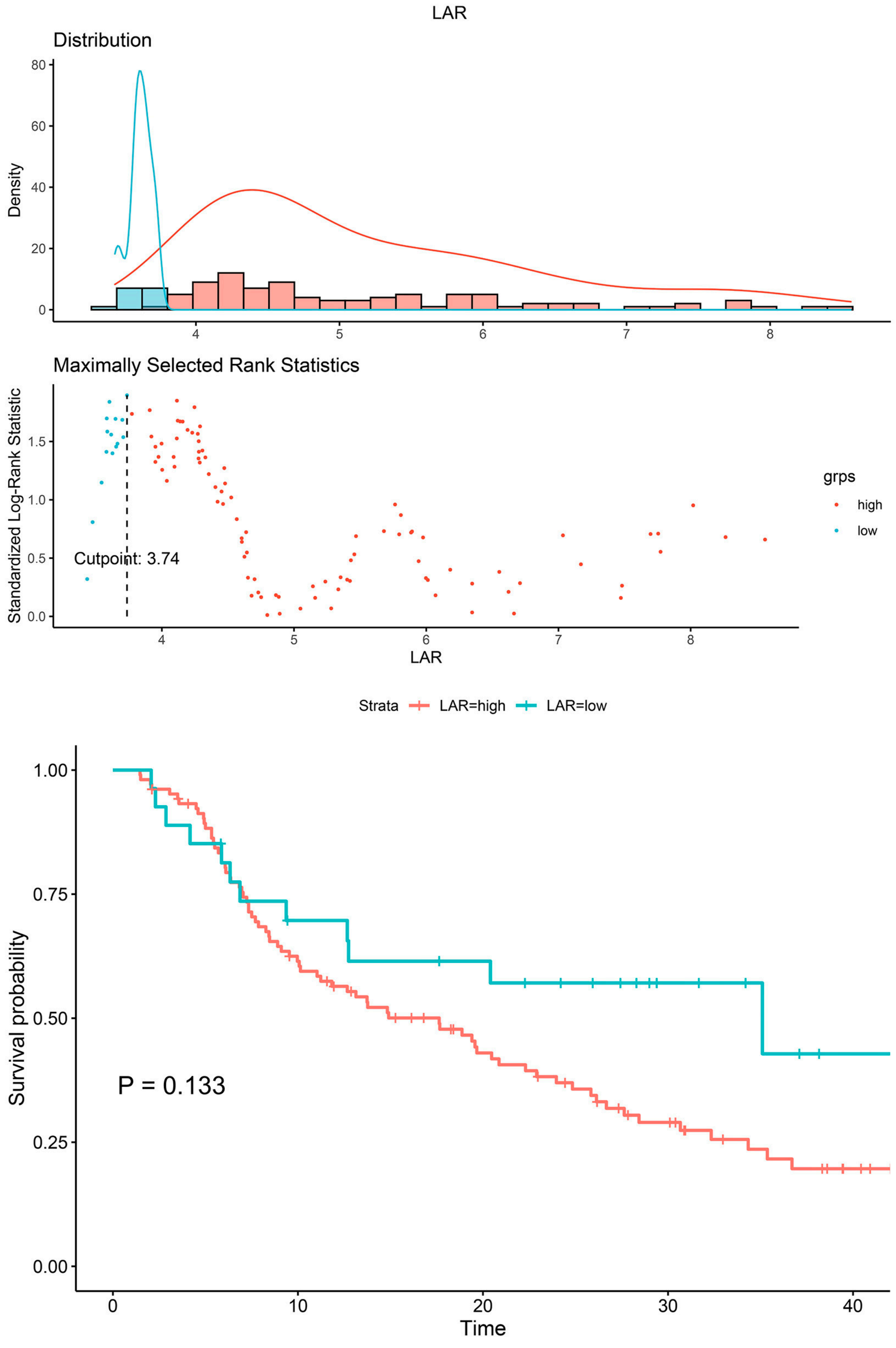

Figure S8. Cutoff of lymphocyte-to-monocyte ratio (LMR) determined by Maximally Selected Rank Statistics, and survival curves stratified by the cutoff.

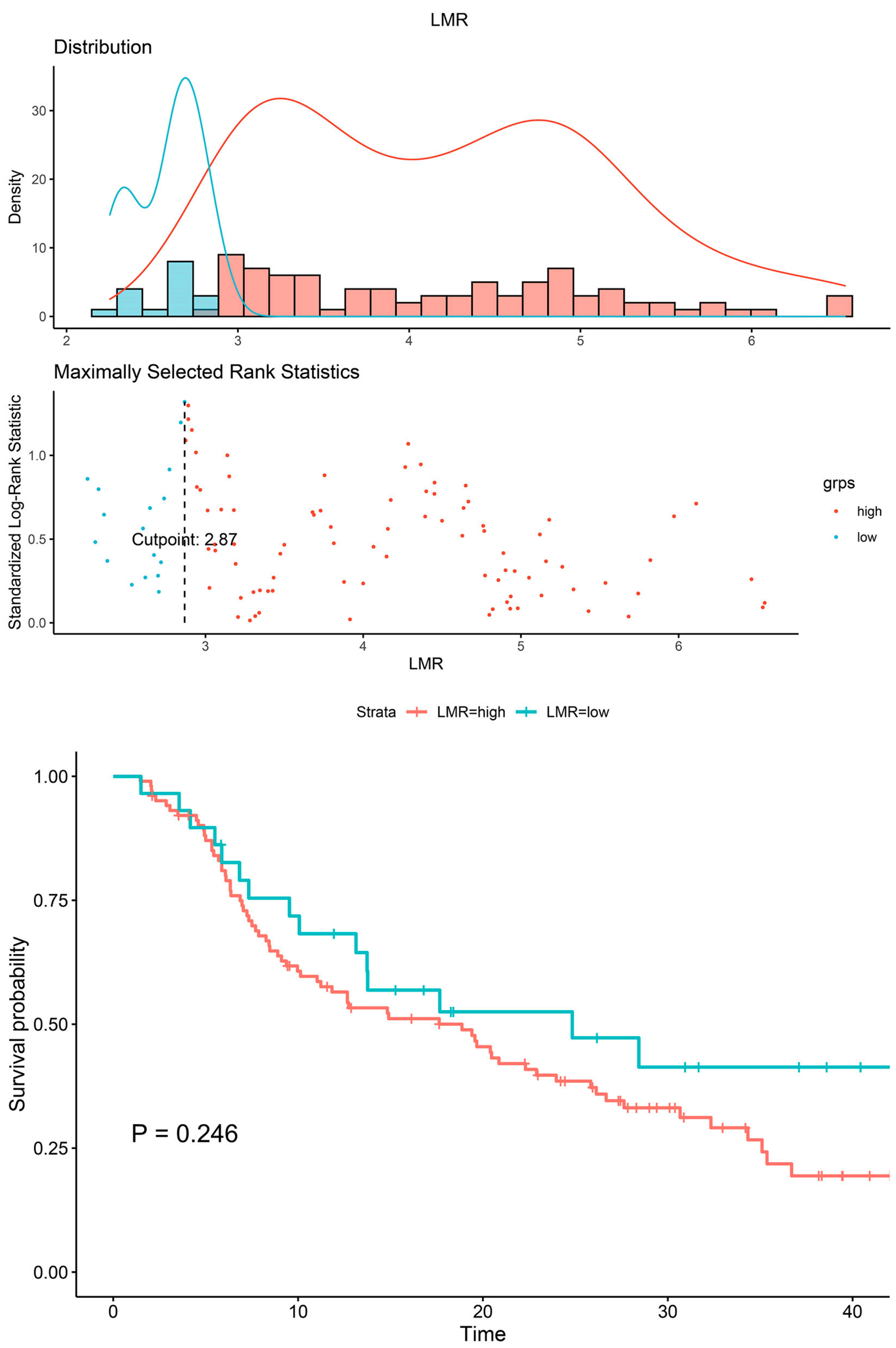

Figure S9. Cutoff of neutrophil-to-Lymphocyte ratio (NLR) determined by Maximally Selected Rank Statistics, and survival curves stratified by the cutoff.

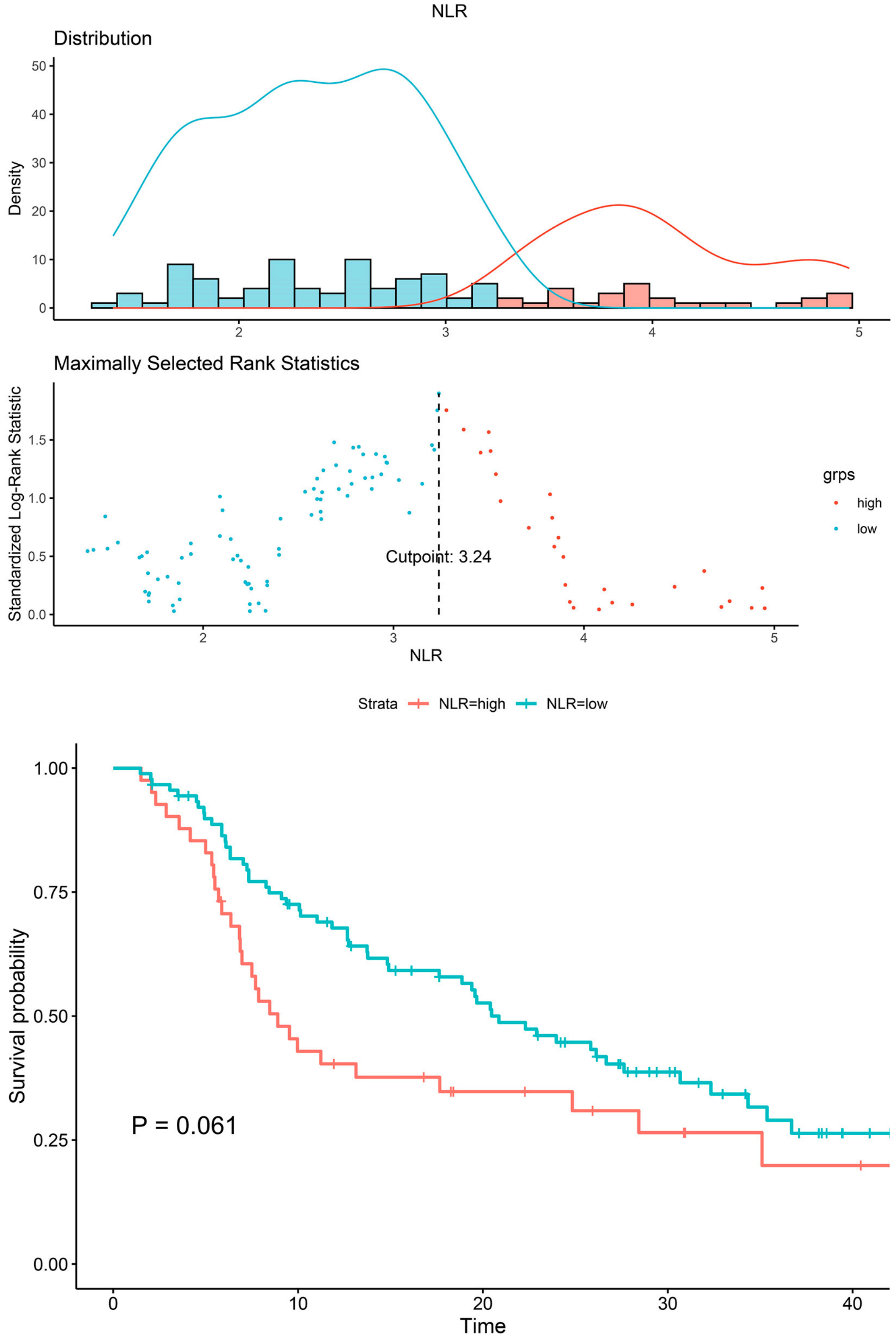

Figure S10. Cutoff of platelet-to-lymphocyte ratio (PLR) determined by Maximally Selected Rank Statistics, and survival curves stratified by the cutoff.

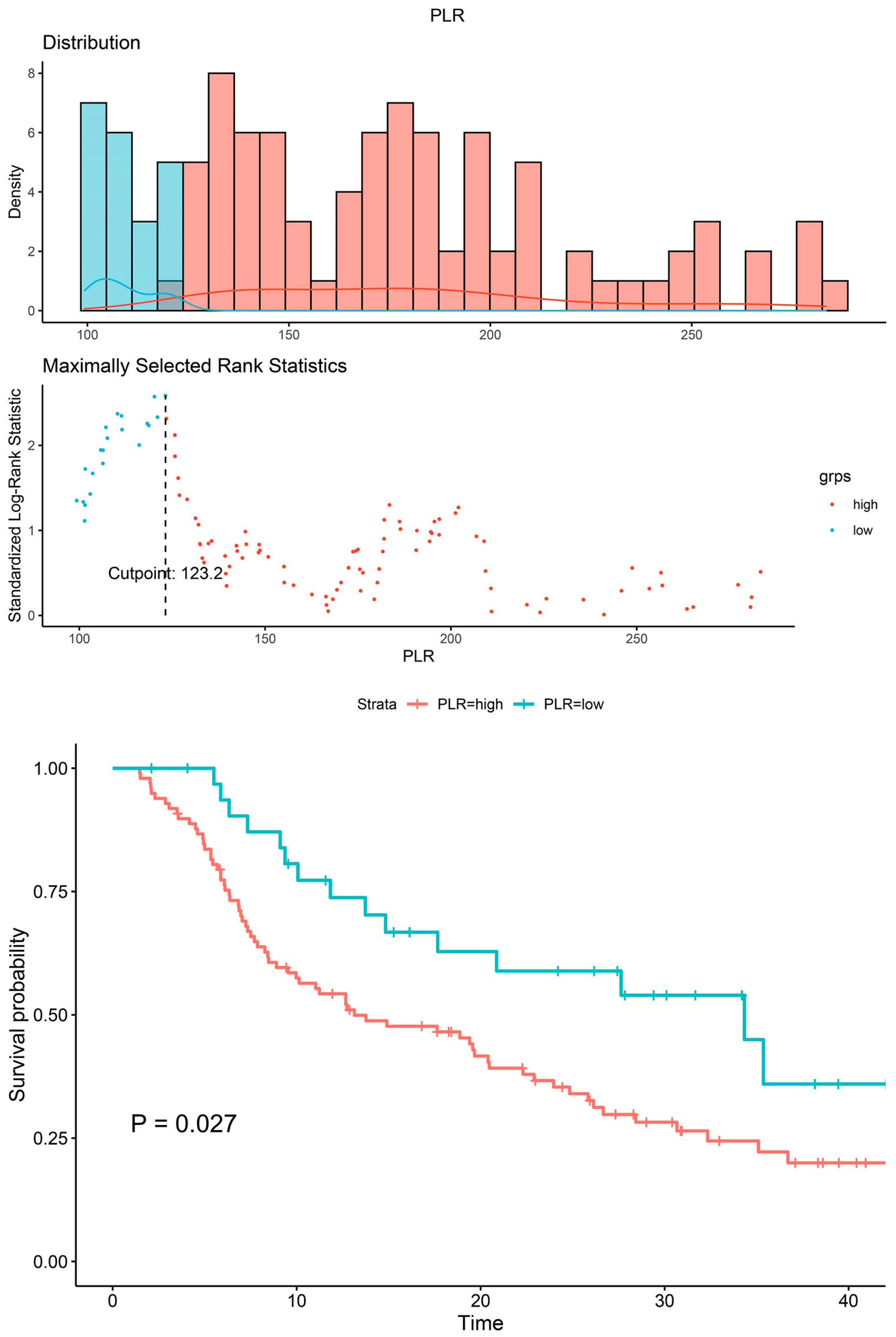

Figure S11. Cutoff of pretreatment Epstein-Barr virus DNA (EBV DNA) determined by Maximally Selected Rank Statistics, and survival curves stratified by the cutoff.

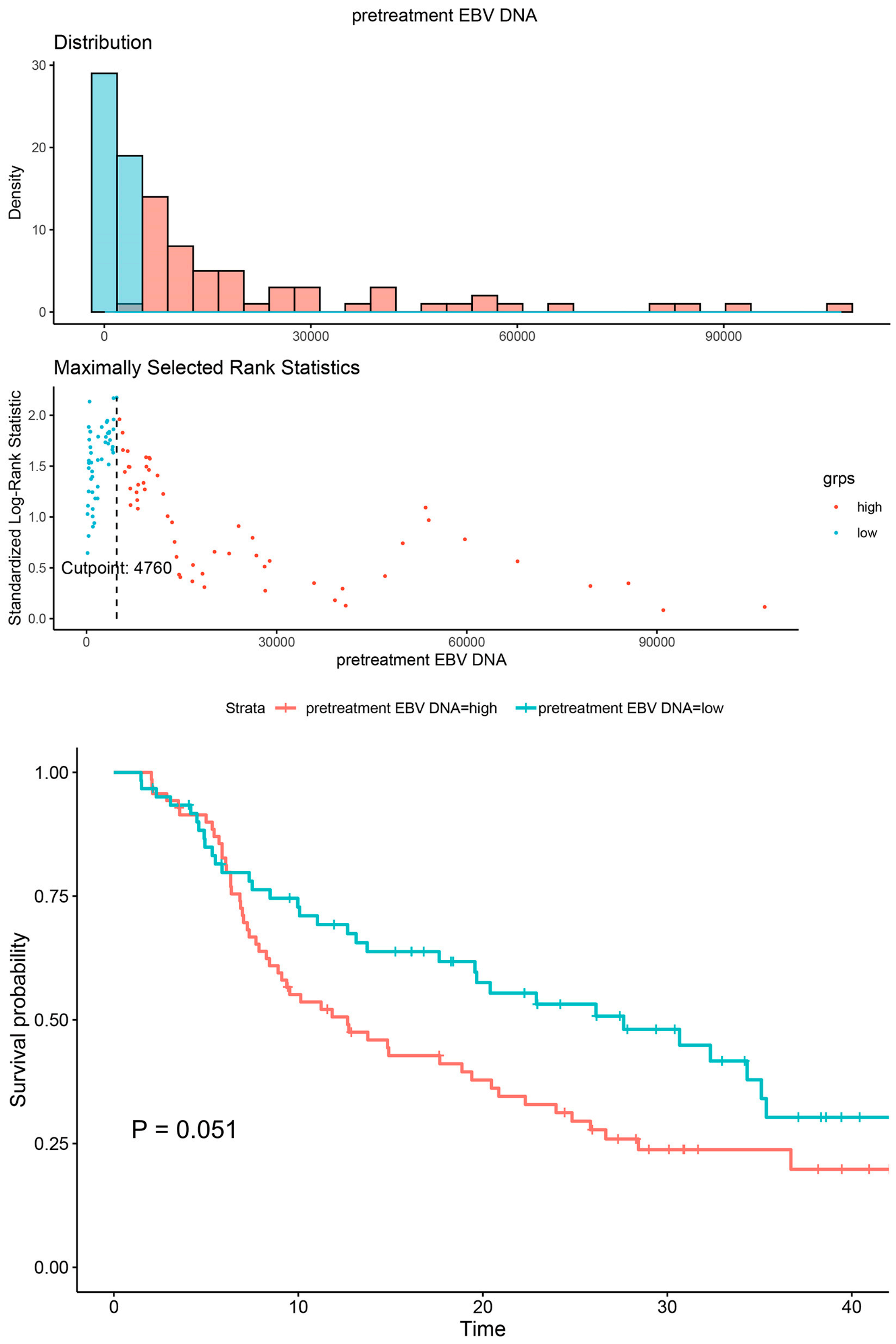

Figure S12. Cutoff of post-treatment Epstein-Barr virus DNA (EBV DNA) determined by Maximally Selected Rank Statistics, and survival curves stratified by the cutoff.

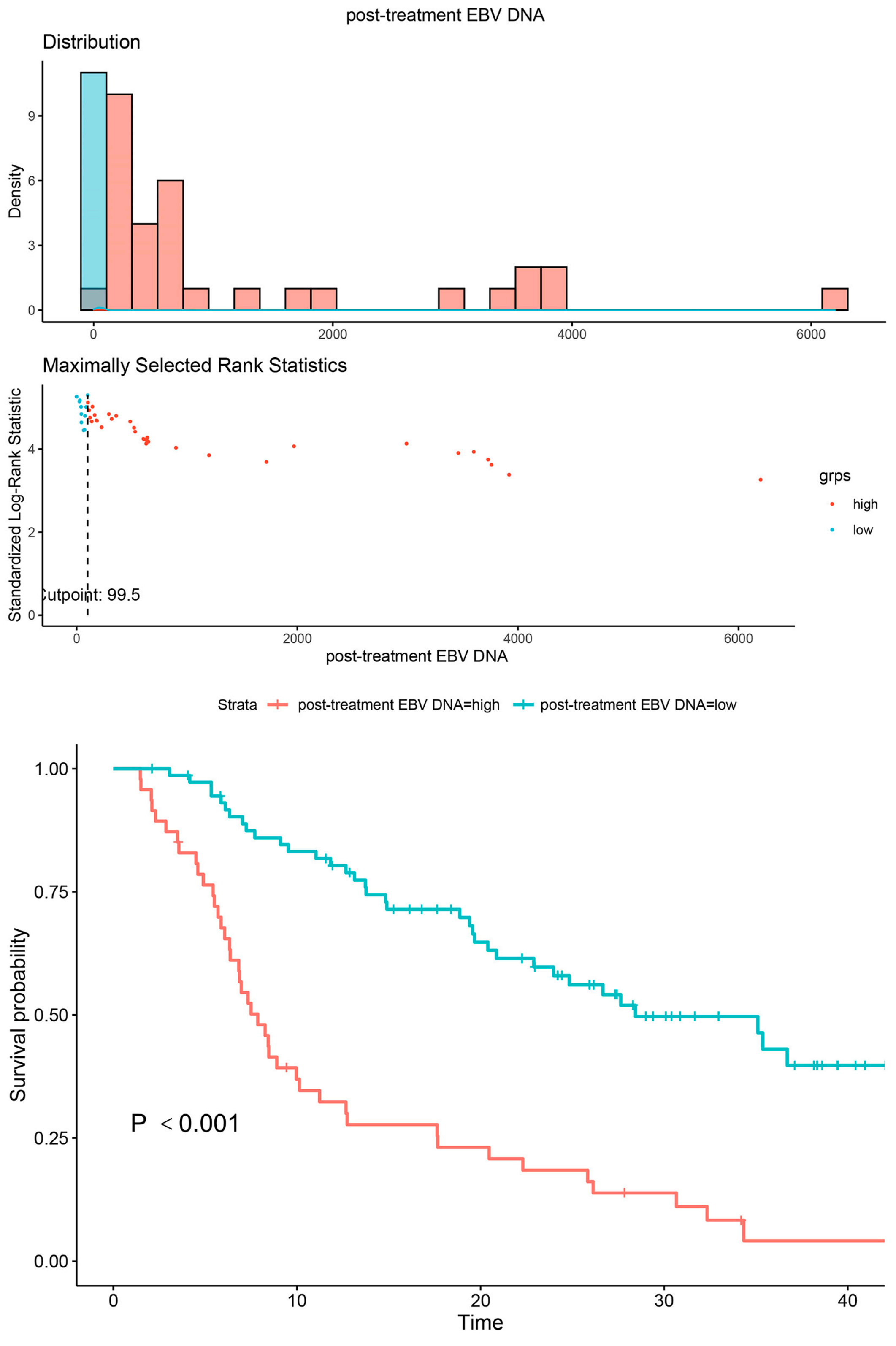

Table S1. Screening tool for Glasgow prognostic score.

Parameter	Values	Score
C reactive protein (mg/L)	≤10	0

	>10	1
Albumin (g/L)	<35	1
	⩾35	0

Table S2. Screening tool for Controlling Nutritional Status score.

**Parameter**	**Malnutrition status**			
	Normal	Light	Moderate	Severe
Albumin (g/L)	⩾35.0	30.0-34.9	25.0-29.9	< 25.0
Score	0	2	4	6
Total lymphocyte count (10^9/L)	⩾1600	1200-1599	800-1199	< 800
Score	0	1	2	3
Total cholesterol (mmol/L)	⩾4.65	3.62-4.64	2.58-3.61	<2.58
Score	0	1	2	3
Total score	0-1	2-4	5-8	9-12
